# Using a Modified Lymphocyte Genome Sensitivity (LGS) test or TumorScan test to detect cancer at an early stage in each individual

**DOI:** 10.1096/fba.1020

**Published:** 2018-12-20

**Authors:** Diana Anderson, Mojgan Najafzadeh, Andrew Scally, Badie Jacob, John Griffith, Rohit Chaha, Richard Linforth, Michel Soussaline, Francoise Soussaline

**Affiliations:** ^1^ School of Life Sciences University of Bradford Bradford UK; ^2^ Faculty of Health Studies University of Bradford Bradford UK; ^3^ Bradford Teaching Hospitals NHS Foundation Trust, Bradford Royal Infirmary Bradford UK; ^4^ IMSTAR SAS Paris France

**Keywords:** cancer, comet assay, lymphocyte genome sensitivity (LGS), tumorScan

## Abstract

Our previous case‐control study observed isolated lymphocytes from 208 individuals and determined the differences in the sensitivity to genomic damage of lymphocytes derived from cancer patients, pre/suspect cancer patients and healthy volunteers using the Comet assay (Anderson et al, 2014). We adapted the LGS technique using a slightly different method and examined 700 more blood samples from 598 patients with cancer or suspected cancer and 102 healthy individuals. To help increase the sensitivity of the test and detect cancer at the level of each individual, we joined with the IMSTAR team who analysed our cells with their fully automated Pathfinder™ cell reader‐analyser system. With this reading and analysis system 4,000 to 10,000 cells were able to be read per slide. The new test which is called TumorScan is a highly sensitive test to detect any cancer at an early stage through the response of the white blood cells to UV treatment. These patient blood samples have also been collected at the stage before confirming diagnosis and treatment. There were four of these individuals with cancer who had received anti‐cancer treatment. The results from these patients showed a reverse pattern compared to non‐treated cancer patients and followed the pattern seen in healthy individuals. The results are consistent with the early results as reported in the above 2014 paper. Given the results from these samples were in a particularly challenging subgroup, whose cancer status was difficult to distinguish, the data suggest that the technique using the TumorScan system could exceed the area under the ROC curve >93% obtained in the earlier study on a group basis, whereas this present study was to detect cancer at an early stage in each individual.

Greater than 360,000 new cancer cases develop in the UK per year, a figure that represents almost 990 cases each day. As such, a cancer diagnosis is made every 2 minutes in the UK.^1^ Also, globally, 14.1 million new cases of cancer are diagnosed every year.^1^ More recently using modern genomic technology. There was the opportunity to further understand the intricacy of interactions between cellular genes and regulatory genetic elements, which are responsible for phenotypes resulting in cancer, in addition to further understanding the complexity of cancer. However, despite these advancements and occasional successes, most treatments are as yet relatively impractical.^2^

According to sources of the national audit and survey data from patients, the tumour site proves the strongest predictor of multiple consultations. Multiple consultations are found in between 30% and 50% of patients subsequently diagnosed with multiple myeloma or pancreatic, stomach, or lung cancer, compared with <10% multiple consultations of patients subsequently diagnosed with breast cancer or melanoma Figure 1.^3^, ^4^ These distinctions appear to reflect the “symptom signature” of various cancers—multiple consultations are less associated with patients of cancers with specific symptoms eg a palpable breast lump or a visible skin lesion), are compared to those who were mostly patients present with non‐specific symptoms eg back or abdominal pain). As such, a marker for the difficulty of detection of a cancer at first presentation may be considered to be the proportion of patients with multiple consultations (Figure 1).^5^

**Figure 1:**
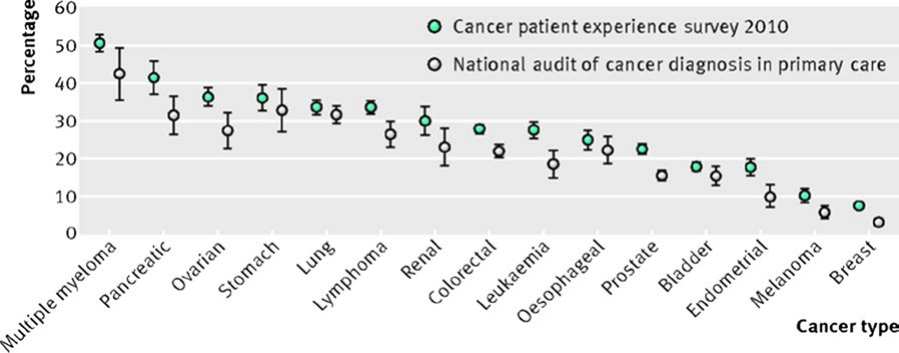
Percentage of patients with cancer who had three or more consultations with a general practitioner before referral. These data can be used to categorise cancers as “easier to suspect” (melanoma, breast, endometrial cancer), “harder to suspect” (multiple myeloma, pancreatic, ovarian, stomach, lung), or of intermediate diagnostic difficulty. Reprinted from Lyratzopoulos G, Neal RD, Barbiere JM, Rubin GP, Abel GA. Variation in number of general practitioner consultations before hospital referral for cancer: findings from the 2010 National Cancer Patient Experience Survey in England. *Lancet Oncol* 2012;13:353‐65 with permission from Elsevier

Therefore, early detection of cancer significantly raises the chances for an effective treatment. Cancer identification methods are greatly based on imaging, biopsies and a few nonspecific biomarkers. The main element of early cancer detection is based on education to assist early diagnosis and screening. There are new blood tests with a high specificity in detecting cancer from healthy individuals.^6^, ^7^, ^8^ However up to the present time, finding an efficient practical method with a low cost and easy approach and use in laboratories over the world was not readily available. However, Anderson et al. 2014^9^ showed detecting genomic damage in lymphocytes from cancer and suspected cancer patient samples compared to healthy individuals using the Comet assay fulfilled this need. The entirety of the cancers tested exhibited responses that were worthy of comparison. The test can categorise an individual as positive or negative for disease based on a threshold value for a continuous variable (Olive Tail Moment^1^ and % Tail DNA^2^). Therefore, an analysis of Receiver Operating Characteristic curves was undertaken based on the 208 individuals. For all cancers, as well as pre/suspected‐cancer, the mean log Olive tail moments compared to controls resulted in values of area under the curve of 0.87 95% CI: 0.82‐0.92). For cancer versus pre/suspected‐cancer plus controls the value was 0.89 95% CI: 0.83‐0.95). Finally, for cancer versus controls, excluding pre/suspected‐cancer, the value was 0.93 95% CI: 0.88‐0.98) for all 3 values *P* < 0.001). The test assessed the susceptibility of the genome to genetic damage. It is very well established that the risk of developing cancer is mostly related to inherited or induced genetic mutations. So by implication, this is an assay that evaluates generic, genomic, genotoxic processes and is thus an empirical assay of cancer susceptibility that does not require an understanding of the underlying causative mechanisms.^9^ In the present study, results indicated that characterisation of differences in lymphocyte sensitivity to UV again enabled discrimination between cancer patients, pre/suspect cancer patients and healthy volunteers. However, since 2012 to facilitate the comet assay method, with UV treatment it was possible to standardise the procedure, and we modified the earlier LGS technique.^10^ The new improved system has been examined on more than 700 individuals eg in Figures 2, 3, 4 and 5. The order of UV treatment was set from lowest and then increasing to the highest UV intensity. The order of UV treatment was set as increasing in intensity, starting initially with the lowest UV intensity.

**Figure 2:**
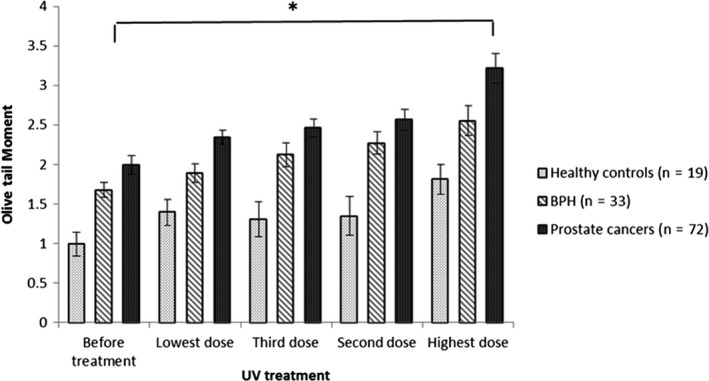
Mean log of the Olive Tail Moment (OTMs) (95% CI) against lymphocyte treated with different doses of UV intensity for all groups. In patients with prostate cancer, there was a constant high level of DNA damage after treating with different UV intensity doses. This contrasted with patterns for healthy individuals, and benign prostate hyperplasia (BPH) with different levels of response to UV treatment. * presents the P‐value <0.01 when the prostate cancer group was compared to the healthy control group

**Figure 3:**
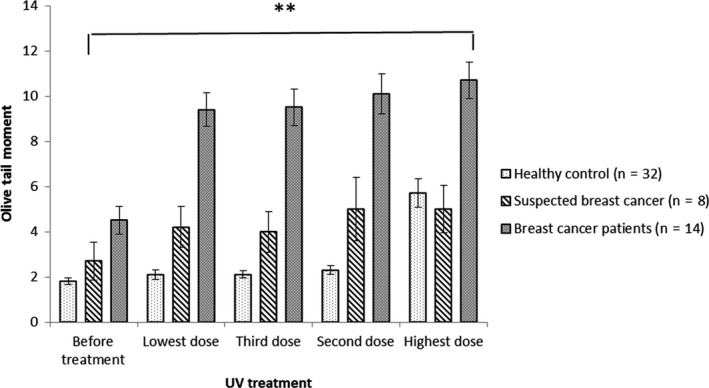
Mean log of the Olive Tail Moment (OTMs) (95% CI) against lymphocytes treated with different doses of UV intensity for all groups. In patients with breast cancer, there was a constant high level of DNA damage after treating with different UV intensity doses. This contrasted with patterns for healthy individuals, and suspected cancer patients (patients with lumps in breast) with different levels of responses to UV treatment. **Presents the *P*‐value <0.05 when the breast cancer group was compared to the healthy control group.

**Figure 4:**
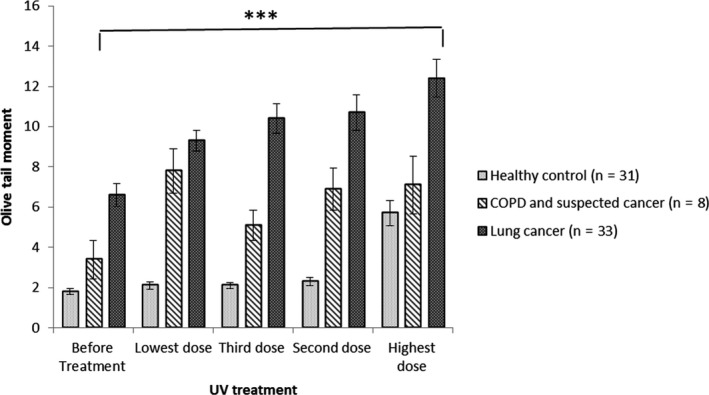
Mean log of the Olive Tail Moment (OTMs) (95% CI) against lymphocytes treated with different doses of UV intensity for all groups. In patients with lung cancer, there was a constant high level of DNA damage after treating with different UV intensity doses. This contrasted with patterns for healthy individuals, and suspected cancer patients (patients with Chronic obstructive pulmonary disease (COPD) with different levels of responses to UV treatment. ***Presents the *P*‐value <0.001 when the lung cancer group was compared to the healthy control group.

**Figure 5:**
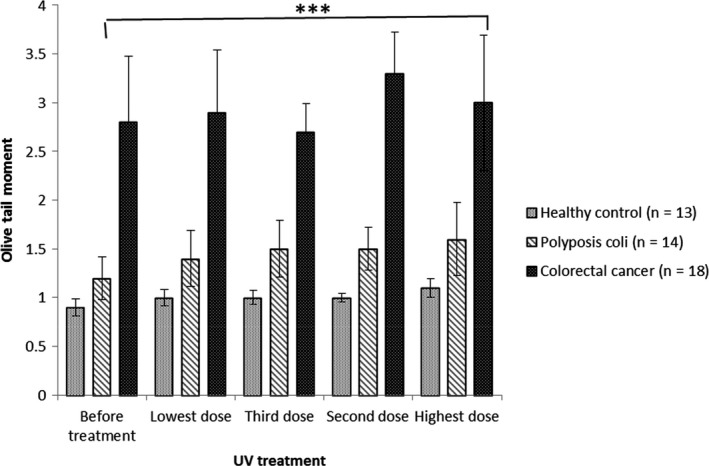
Mean log of the Olive Tail Moment (OTMs) (95% CI) against lymphocytes treated with different doses of UV intensity for all groups. In patients with colorectal cancer, after treating with different UV intensity doses there was a constant high level of DNA damge. This contrasted with patterns for healthy individuals, and suspected cancer patients (patients with polyposis coli) with different levels of responses to UV treatment. ***Presents the *P*‐value <0.001 when the colorectal cancer group was compared to the healthy control group.

The original scoring system was performed using the semi‐automated fluorescent Olympus microscope and Komet 6 software. The number of scored cells was 100 per dose.^9^, ^10^ There were some individuals with cancer and suspected cancer and healthy controls that did not follow the patterns in the damage of DNA in the other samples from the same groups Figure 6. These were samples which were difficult to distinguish the disease state by comparison with healthy controls.

**Figure 6:**
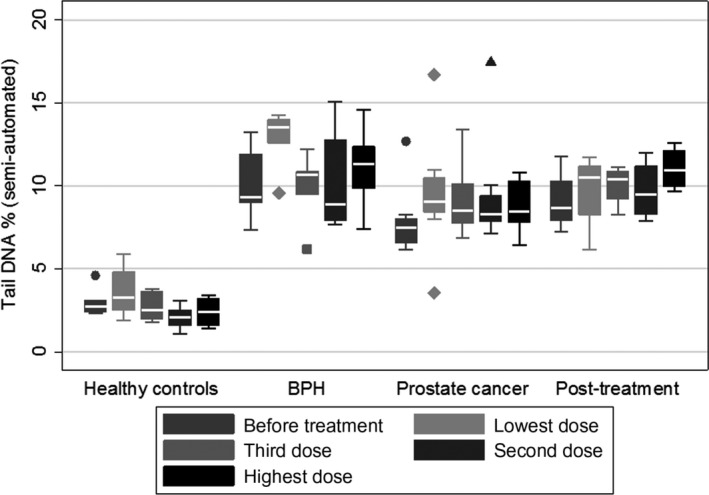
Prostate group: semi‐automated scoring system (difficult to distinguish samples). Group 1 = healthy control, group 2 = benign prostate hyperplasia, group 3 = prostate cancer and group 4 = prostate cancer after treatment

Sharma et al showed the sensitivity of the in vitro comet assay increases with the number of cells scored, for low levels of DNA damage, eg scoring of 600 cells increased the sensitivity compared with scoring of 100 cells with statistical significance. Therefore, in their test it sensitivity can be improved by scoring more than 100 cells, using the fully‐automated Comet assay scoring.^11^ The new collaboration which includes the modified comet assay plus UV treatment (University of Bradford) and the fully‐automated Comet assay scoring (Pathfinder^TM^ Comet‐imaging system from IMSTAR, Paris, France) validated by Jackson et al.^12^ is called TumorScan. We investigated the difficult to distinguish samples from two groups of studies, prostate and colorectal projects, with both the semi‐automated and fully‐automated scoring systems. The sample of pre‐cancer and cancer patients in the prostate group: healthy control n = 10, BPH n = 5, prostate cancer n = 10 and in the colorectal group: healthy control n = 13, polyposis coli n = 10, colorectal cancer n = 21) selected for reanalysis here represent a subset of cases where the semi‐automated system could not distinguish between patients with cancer and those with pre‐cancerous conditions. The sample therefore represents the particularly challenging cases (Figures 6, 7 and 8).

**Figure 7:**
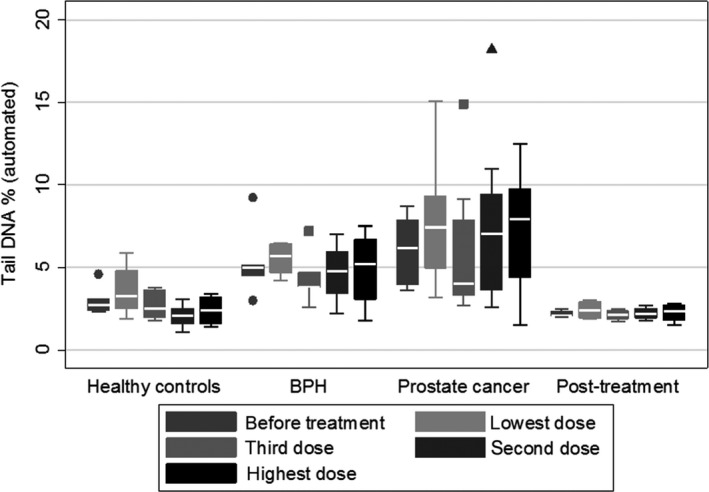
Prostate group, automated scoring system (difficult to distinguish samples). Group 1 = healthy control, group 2 = BPH, group 3 = prostate cancer and group 4 = prostate cancer after treatment

**Figure 8:**
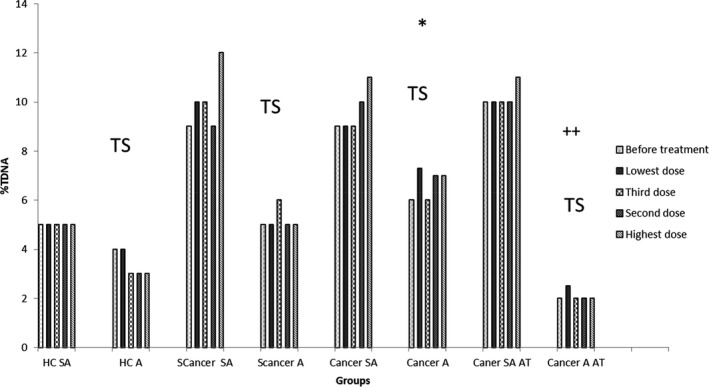
Mean log of the %Tail DNA against lymphocytes treated with different doses of UV intensity for all groups. In patients with colorectal and prostate cancer, after treating with different UV intensity doses compared to healthy individuals, and suspected cancer patients (patients with polyposis coli and BPH) with different levels of responses to UV treatments. Also, the comparison of two various semi‐automated and automated (TumorScan) (TS) reading system in colorectal and prostate study groups. (HC = healthy control, SA = semi‐automated, A = automated, Scancer = suspected cancer, AT = after treatment). *Represents the *P*‐value <0.05, when healthy controls group compared to cancer group in the automated system and ++ represents the *P*‐value <0.05 when cancer after treatment group in the automated system compared to cancer after treatment group in the semiautomated system.

For both Olive Tail Moment (OTM) and %Tail DNA (TDNA), the mean values in the pre‐cancer group are higher than in the cancer group for this selective sub‐sample when using the original analysis method.

In the fully‐automated system, data showed the average values of both OTM and TDNA are higher in pre‐cancerous conditions, compared to controls; they are higher still in cancer patients, but the difference between mean values for the pre‐cancer and cancer groups is not statistically significant in this small sample OTM: *P* = 0.282; TDNA: *P* = 0.158). There is a statistically significant difference between the cancer group and the control group *P* = 0.007 and *P* < 0.001, for OTM and TDNA, respectively) (Figures 7 and 8).

The data that we presented are the samples that were difficult to discriminate between healthy controls and cancer patients. The numbers of investigated samples for each group are stated in the text. This research was designed as a case‐control study as in the 2014 study and there was a 10‐15 years gap between the healthy control group and cancer groups.  The confounding factors smoking, age, ethnicity, drinking habit and gender) for each cancer group lung cancer, colorectal cancer, prostate, and breast cancers) were carried out using the T‐test as shown in Tables 1, 2, 3, and 4. Results shown that, there were no significant differences in any of the confounding factors. For breast cancer, only female were considered. See the data collection form in Appendix 1.

**Table 1:** Confounding factors in lung cancer study

Confounding factors	*P* value
Smoking	<0.902
Age	<0.354
Ethnicity	<0.800
Drinking habit	<0.21
Gender	<0.178

**Table 2:** Confounding factors in colorectal cancer study

Confounding factors	*P* value
Smoking	<1.08
Age	<0.205
Ethnicity	<0.902
Drinking habit	<1.003
Gender	<0.20

**Table 3:** Confounding factors in prostate cancer study

Confounding factors	*P* value
Smoking	<0.95
Age	<0.307
Ethnicity	<1.401
Drinking habit	<0.103
Gender	N/A

**Table 4:** Confounding factors in breast cancer study

Confounding factors	*P* value
Smoking	<1.05
Age	<0.403
Ethnicity	<1.02
Drinking habit	<0.108
Gender	N/A

For the cancer group who have received cancer treatment, OTM and TDNA both returned to normal levels, (Figures 7 and 8).

The results are consistent with the early results reported in the Anderson et al paper and, given these results are in the very challenging subgroup, the data suggest that the technique using the fully automated scoring system may even exceed the area under ROC curve >93% obtained by the early LGS system, if in any population there will be blood samples of individuals for whom it will be difficult to predict the cancer status.

As pointed out by Anderson et al,^9^ it remains an open question whether the increase in damage in cancer cells is a predictor of susceptibility or a consequence of disease. We know that therapeutics are not involved in this study since patients samples were acquired pre‐diagnosis and thus pre‐treatment. It was only after obtaining the pathology report from the consultant at the clinic that cancer or other disease states could be confirmed. It is possible that both susceptibility and cancer status could be involved but this can only be ascertained by a long‐ term cohort study over several decades or in a mortality and morbidity study with pooled data bases linked to statistics from cancer registries. In terms of identifying those at risk of having or developing cancer, this uncertainty has no effect on the functionality of the assay because of its empirical nature and the power of the assay is based on this concept. In 2012, Najafzadeh et al suggested that by comparing negative control responses with high UVA exposure responses, differences in individual cancer risk could be considered. However, by incorporating responses at different depths in the agar or at different light intensities discrimination is enhanced by gaining information about varied UVA intensity‐dependent responses.^9^

Since that 2014 study, we have conducted two clinical trials, one on colon cancer and the other on prostate cancer and samples from a few others eg breast, and the data were examined in a similar way as in the earlier study bringing our total individuals to 908 of whom 196 were controls. In this second tranche since 2014, 60 patients were difficult to distinguish from controls. It was then that we teamed up with IMSTAR for slide scoring. We have worked from the endpoint backwards in our own validation, since we have had the cancer patient status confirmed by the relevant consultant in the clinics. Those samples that belong to other disease states are used in research studies with this system. Since this tranche is concerned with this new study, where we are predicting at an early a stage as possible, the difference between cancer patients and controls, we approached IMSTAR to determine if their automated system might help since large numbers of cells can be scored within minutes with no human intervention, thereby increasing the sensitivity, and as in 2014 there is no need for extra enzymes to increase sensitivity.

This final study confirms that the modified method, TumorScan ‐ which combines the adapted Comet assay with different UV treatments, designed and fully automated cell reader‐analyser (IMSTAR Pathfinder^TM^), successfully improves the sensitivity of the original test (LGS test) for the detection of samples at the level of the individual.

## DISCLOSURES

The authors report no conflicts of interest.

## AUTHOR CONTRIBUTIONS

Diana Anderson: Joint first author, Principle Investigator; Mojgan Najafzadeh: Joint first author, researcher on this project; Andrew Scally: Statistician; Badie Jacob: Respiratory Consultant, assisting in collecting blood samples from patients with pulmonary diseases; John Griffith: Colorectal Consultant, assisting in collecting blood samples from patients with colorectal diseases; Rohit Chaha: Urology Consultant, assisting in collecting blood samples from patients with prostate diseases; Richard Linforth: Breast cancer Consultant, assisting in collecting blood samples from patients with suspected breast cancer; Michel Soussaline: collection of data from Pathfinder; Francoise Soussaline: collection of data from Pathfinder.

## ENDNOTES

1 Olive Tail Moment is defined as product of distance and normalized intensity integrated over the tail length. A damage measure combining the amount of DNA in the tail with distance of migration (severity of damage).

2 Percentage of DNA in Tail is the integrated tail intensity x 100 divided by the total integrated cell intensity for a normalized measure of the percent of total cell DNA found in the tail.
